# Genetics of Growth Reaction Norms in Farmed Rainbow Trout

**DOI:** 10.1371/journal.pone.0135133

**Published:** 2015-08-12

**Authors:** Panya Sae-Lim, Han Mulder, Bjarne Gjerde, Heikki Koskinen, Marie Lillehammer, Antti Kause

**Affiliations:** 1 Aquaculture and Genetics, Nofima, Osloveien 1, Ås, Norway; 2 Animal Breeding and Genomics Centre, Wageningen University, Wageningen, the Netherlands; 3 Aquaculture Unit, Natural Resources Institute Finland, Tervo, Finland; 4 Biometrical Genetics, Natural Resources Institute Finland, Jokioinen, Finland; Temasek Life Sciences Laboratory, SINGAPORE

## Abstract

Rainbow trout is farmed globally under diverse uncontrollable environments. Fish with low macroenvironmental sensitivity (ES) of growth is important to thrive and grow under these uncontrollable environments. The ES may evolve as a correlated response to selection for growth in one environment when the genetic correlation between ES and growth is nonzero. The aims of this study were to quantify additive genetic variance for ES of body weight (BW), defined as the slope of reaction norm across breeding environment (BE) and production environment (PE), and to estimate the genetic correlation (*r*_*g*(int, sl)_) between BW and ES. To estimate heritable variance of ES, the coheritability of ES was derived using selection index theory. The BW records from 43,040 rainbow trout performing either in freshwater or seawater were analysed using a reaction norm model. High additive genetic variance for ES (9584) was observed, inferring that genetic changes in ES can be expected. The coheritability for ES was either -0.06 (intercept at PE) or -0.08 (intercept at BE), suggesting that BW observation in either PE or BE results in low accuracy of selection for ES. Yet, the *r*_*g*(int, sl)_ was negative (-0.41 to -0.33) indicating that selection for BW in one environment is expected to result in more sensitive fish. To avoid an increase of ES while selecting for BW, it is possible to have equal genetic gain in BW in both environments so that ES is maintained stable.

## Introduction

The performance of organisms is influenced by the surrounding environmental conditions, leading to phenotypically plastic responses to environmental changes. Such plastic responses have been observed, for example, as adaptive plasticity in the neck teeth of *Daphnia* (water fleas) which develops as a protective response to the chemical cues of a predatory *Chaoborus* present in the water [1]. In fish species, phenotypic plasticity has been explored especially from ecological and evolutionary points of view. There is evidence for genetic basis of phenotypic plasticity, for example in salmonids [2], Trinidadian guppies (*Poecilia reticulata*) [3,4] and pupfishes (*Cyprinodon nevadensis*) [5].

Among animal breeders, phenotypic plasticity is termed macroenvironmental sensitivity (ES) [6]. It has been of interest for animal breeders because of its connection with animal’s performance across environments [7,8] and to the robustness and welfare of animals [9]. For a genotype, such as a clone, family, population, or a species, macroenvironmental sensitivity can be defined by its slope of reaction norm across environments. Assuming a linear reaction norm, the degree of macroenvironmental sensitivity can be quantified by the regression slope of a genotype's performance, such as growth, against an environmental gradient [10–12].

Rainbow trout *Oncorhynchus mykiss* (Walbaum 1792) is one of the main fish species farmed under diverse environmental conditions across continents. Rapid growth is one of the most important traits for profitable trout farming. However, fish may not be able to maintain high growth when rearing conditions are suboptimal. Therefore, a more robust fish with high stability of growth is important to thrive under variable environmental conditions. To quantify the potential for changing macroenvironmental sensitivity through selection, an estimate of genetic variance in macroenvironmental sensitivity is required. The genetic variation in the macroenvironmental sensitivity is known as non-parallel reaction norms, causing genotype-by-environment interaction (GxE) [13]. Evidences of GxE in growth of rainbow trout have been reported [14–20]. However, so far most studies use multi-trait model in which GxE is quantified as the genetic correlation between the records of the same trait measured in different environments. Such genetic correlation expresses the magnitude of re-ranking of families with respect to their breeding value, but it does not provide an explanation on how macroenvironmental sensitivity can evolve across environments. The concept of macroenvironmental sensitivity has never been applied to breeding in aquaculture before.

In Finland, the national breeding programme for rainbow trout breeds especially for improved growth performance in commercial production environment at the Baltic Sea [21]. However, the stock is also reared in inland freshwater production environments, and exported to Russia and Asia where the production environment differs from Finland substantially. Hence, the macroenvironmental sensitivity is considered as an important trait.

The aims of this study were two-fold. Firstly, we quantify the genetic variance for ES, defined as the slope of reaction norm across seawater and freshwater production environments in Finland, using a reaction norm model. Secondly, to study whether selection for fast growth in one environment will change ES, we estimate the genetic correlation between ES and body weight in one environment, defined as the intercept of reaction norm. In addition, we derived the coheritability for ES. Although, the genetic covariance matrix from reaction norm and multi-trait models is interchangeable [7,22–23], the reaction norm model is chosen as the method in this study because it provides the genetic parameters for ES and body weight directly without interchanging. To be able to compare our results with previous studies, we exploit this interchangeable property to calculate genetic variance in ES and its genetic correlation with intercept in aquaculture GxE studies that all have used a multi-trait model.

## Materials and Methods

### Ethics Statement

All procedures involving animals were approved by the animal care committee of the Natural Resources Institute Finland. To enhance animal welfare and ameliorate suffering during all fish handling, the fish were always first anaesthetized using MS-222.

### Data source

All fish used in this study were obtained from the Finnish national breeding programme. Breeding candidates are held at the Tervo fish farm in central Finland (freshwater nucleus station) and the sibs of the breeding candidates are tested at commercial sea stations located at the Baltic Sea. The phenotypic data had 53,638 records of body weight at tagging from four year classes and belonged to two subpopulations, one with year classes of 1996 and 1999 and the other with 1997 and 2000. Both of these subpopulations were established from the parents of year class 1993. Sires were mated to dams using either paternal nested mating or partial factorial mating designs. Each year class consisted of 94 to 197 full-sib families established from the mating of 37 to 95 sires with 79 to 129 dams. After hatching, fingerlings from the same full-sib family were held in one or more family tanks until the fingerlings reached tagging size (mean body weight of approximately 50 g).

During the tagging, full-sibs from each family were randomly sampled and divided into two or three batches that were reared either at the freshwater nucleus station (defined as “breeding environment” or BE) or at one or two seawater stations (defined as “production environment” or PE) at the Baltic Sea. When the fish were 2-year-old, they were individually weighed at BE (trait: BW_BE_, in g) and PE (trait: BW_PE_, in g) stations. The total number of records analysed was 22,175 individuals for BW_BE_ and 20,865 individuals for BW_PE_ (Table 1). The average BW_BE_ (SD) and BW_PE_ (SD) were 1094 (363.9) g. and 1050.0 (334.5) g, respectively. The pedigree was traced back to the parents (the founders) of the 1990 year class. The ancestors back to the founder population of the 1990 year class were included in the pedigree.

**Table 1:** Population structure.

	Subpopulation I		Subpopulation II	
	1996	1999	1997	2000
*Population structure*				
Sires, dams	57, 129	37, 94	65, 79	95, 121
Sires per dam, mean (range)	1.00 (1–1)	1.00 (1–1)	2.41 (1–3)	1.63 (1–3)
Dams per sire, mean (range)	2.26 (1–4)	2.54 (1–4)	2.93 (1–5)	2.06 (1–5)
Full-sib families, family tanks	129, 129	94, 135	191, 259	197, 197
*Number of fish with records*				
Freshwater nucleus station	4994	3084	8099	5998
Fish per full-sib family	38.7	32.8	42.4	30.4
Seawater station	2573	2442	8351	7499
Fish per full-sib family	19.9	26.0	43.7	38.1

### Genetic Analysis

#### Reaction norm model

A reaction norm model was used to estimate genetic (co)variance for ES (regression slope) for body weights recorded on 2-year-old fish. (Co)variance components of all analyses were estimated using restricted maximum likelihood in ASReml version 3.0 [24]. Approximate standard errors were calculated with ASReml following Fisher et al. [25].

In addition to the analysis of observed body weights, the analysis was also performed with log-transformed body weights. This was to test the hypothesis that genetic variance in ES may be influenced by a scale effect, typically observed for body weight in fish species, i.e., increasing variance for BW with increasing mean for BW. For instance, parallel reaction norms for genotypes (no genetic variance for slopes) with different intercepts are in fact translated into different magnitudes of sensitivity if change in body weight is calculated as a percentage change in the trait mean. The log-transformation reduces such scale effect [26].

The reaction norm model was:

$$\begin{array}{l} y_{hijklmn}=\beta_{\text{int}}+\beta_{\text{sl}}X_{h}+YC\times SITE\times SEX\times MAT_{ijkl}+\\ \;a_{m,\text{int}}+a_{m,\text{sl}}X_{h}+c_{n,\text{int}}+c_{n,\text{sl}}X_{h}+e_{hijklmn}, \end{array}$$ (1)

where *y* is the observation (body weight or log body weight) of the *m* ^th^ individual. The *β*_int_ and *β*_sl_ are the fixed regression coefficients for the population intercept (int) and slope (sl), respectively. The *X*_*h*_ is the regressor for the environments (*X*_*h*_ = 0 and 1) in which the intercept was placed at *X*_*h*_ = 0. The fixed effect *YC*×*SITE*×*SEX*×*MAT* was included in the model to correct for the interaction of the *i* ^th^ year class (*YC*, *i* = 1996, 1997, 1999, 2000), the *j* ^th^ test station (*SITE*, *j* = 1: BE, 2 to 4: sea-test stations), the *k* ^th^ sex (*SEX*, *k* = 1: male, 2: female, or 9: unknown), and the *l* ^th^ maturity (*MAT*, *l* = 2: mature at 2-year-old, 3: mature at 3-year-old, 9: unknown). The *a* is the random additive genetic effect of intercept (int) and slope (sl) of reaction norm, $\left[\begin{array}{c} a_{\text{int}}\\ a_{\text{sl}} \end{array}\right]$ ~ MVN[**0**, **A**⊗**G**_RN_], where **A** is the additive genetic relationship matrix, **G**_RN_ is genetic covariance matrix from the reaction norm model, and MVN is multivariate normal distribution. The *c*_*n*_ is the random full-sib tank effect (unique numbers in different year classes), explaining an effect common to full-sibs other than additive genetics (tank effect due to the separate rearing of the families prior to tagging and non-additive genetic effect), $\left[\begin{array}{c} c_{\text{int}}\\ c_{\text{sl}} \end{array}\right]$ ~ MVN[**0**, **I**⊗**C**_**RN**_], where **C**_**RN**_ is common environmental covariance matrix and **I** is the identity matrix. The *e*~ N(**0,** $\left[\begin{array}{cc} I\sigma_{e_{1}}^{2} & 0\\ 0 & I\sigma_{e_{2}}^{2} \end{array}\right]$) is the random residual effect of an animal *m* in environment *h* with for each environment a different residual variance. The sire’s and offspring’s estimated breeding values (EBVs) for slope obtained from model (1) were used to illustrate the range of additive genetic values of slope available for selection.

The magnitude and the sign of a genetic correlation between the slope and intercept, and genetic variance for the intercept, can change depending on which environment the intercept is defined. Hence, the model was run twice, either with PE ($X_{h_{1}}$ = 0) or BE ($X_{h_{2}}$ = 0) as the intercept environment. To illustrate the covariance between EBVs of slope and intercept, sire’s EBVs for the slope when the intercept were placed at PE were ranked and a total of fifteen sires with the highest, close to zero, and the lowest EBVs for the slope were chosen for plotting the reaction norm.

#### Genetic characteristics of macroenvironmental sensitivity

The strict sense of heritability for ES is the ratio between additive genetic variance of a slope to phenotypic variance of the slope. Due to the lack of phenotypic variance of the slope, it is not possible to calculate the heritability for ES. Three alternative parameters were used here to describe genetic characteristics of ES. Following Scheiner [27], heritability for ES ($h_{ES}^{2}$) was calculated as:

$$h_{ES}^{2}=\frac{\sigma_{GxE}^{2}}{\sigma_{P,ANOVA}^{2}}\;,$$ (2)

where $\sigma_{GxE}^{2}$ is genotype by environment interaction variance. The $\sigma_{GxE}^{2}$ is equal to the standardized additive genetic variance of the slope (${\hat{\sigma }}_{a,\text{sl}}^{2}\times {\hat{\sigma }}_{X}^{2}$) that is independent from different scales of an environmental variable (*X*). The ${\hat{\sigma }}_{X}^{2}$ is the variance of *X* [28], i.e., ${\hat{\sigma }}_{X}^{2}$ is 0.5 in this study, as the possible values of *X* in this study are 0 and 1. The $\sigma_{P,ANOVA}^{2}$ is total phenotypic variance across environments, in Scheiner's approach calculated from an analysis of variance (ANOVA) [27]. Because $\sigma_{P,ANOVA}^{2}$ may not be available from the reaction norm model, we adopted Scheiner’s heritability by replacing the $\sigma_{P,ANOVA}^{2}$ by ${\hat{\sigma }}_{P,Total}^{2}=\left[\frac{\left(n_{\text{BE}}-1\right){\hat{\sigma }}_{P_{\text{BW},\text{BE}}}^{2}+\left(n_{\text{PE}}-1\right){\hat{\sigma }}_{P_{\text{BW},\text{PE}}}^{2}+n_{\text{BE}}n_{\text{PE}}{\left({\bar{W}}_{\text{BE}}-{\bar{W}}_{\text{PE}}\right)}^{2}/(n_{\text{BE}}+n_{\text{PE}})}{\left(n_{\text{BE}}+n_{\text{PE}}-1\right)}\right]$, where *n* is the number of animals with a record for an animal trait, ${\hat{\sigma }}_{P_{\text{BW}}}^{2}$ is phenotypic variance of the trait and $\bar{W}$ is the mean. Note that neither $\sigma_{P,ANOVA}^{2}$ nor ${\hat{\sigma }}_{P,Total}^{2}$ is the phenotypic variance of the environmental sensitivity. Thus, $h_{ES}^{2}$ is more descriptive rather than a predictive parameter [28]. Furthermore, the definition of heritability in Eq (2) does not coincide with the heritability being the regression of breeding value on phenotype.

Because the expression in Eq (1) is not predictive for response to selection, we defined a second measure called coheritability following selection index principles. The phenotype (*P*) of an individual that includes reaction norm parameters can be defined as (1). We assume no covariances among *a*, *c*, and *e*, because there is no relationship among *a*, *c*, and *e*. The phenotypic variance ($\sigma_{P}^{2}$) of a trait is:

$$\sigma_{P}^{2}=\sigma_{a,\text{int}}^{2}+2X\sigma_{a,\text{int},\text{sl}}+X^{2}\sigma_{a,\text{sl}}^{2}+\sigma_{c,\text{int}}^{2}+2X\sigma_{c,\text{int},\text{sl}}+X^{2}\sigma_{c,\text{sl}}^{2}+\sigma_{e}^{2}$$ (3)

Estimated additive genetic effect of slope (${\hat{a}}_{\text{sl}}$) is equal to the regression on *P* deviated from the population mean, or ${\hat{a}}_{\text{sl}}=b(P-\mu )$. The regression coefficient (*b*) of the breeding value of slope on phenotype is:

$$b=\frac{\text{cov}(a_{\text{sl}},P)}{\sigma_{P}^{2}}=\frac{\sigma_{a,\text{int},\text{sl}}+X\sigma_{a,\text{sl}}^{2}}{\sigma_{P}^{2}}$$ (4)

The *b* in Eq (4) is “coheritability” for ES. The term coheritability is used instead of heritability because coheritability defines the inheritance of association between ES and BW in one environment. The additive genetic covariance between intercept and slope changes along the levels of the environmental factor (*X*). Hence, the magnitude and sign of coheritability is dependent on the value of *X*. A negative coheritability is possible if the absolute of −*σ*_*a*,int, sl_ is greater than $X\sigma_{a,\text{sl}}^{2}$ and/or *X* is negative and absolute $X\sigma_{a,\text{sl}}^{2}$ is greater than *σ*_*a*,int, sl_. The sign of the coheritability explains the change in correlated response of ES when mass selection for higher phenotypic values is performed. When intercept is placed to the environment, in which selection is practised on *P*, *X* becomes zero, leading to:

$$b=\frac{\sigma_{a,\text{int},\text{sl}}}{\sigma_{a,\text{int}}^{2}+\sigma_{c,\text{int}}^{2}+\sigma_{e,\text{int}}^{2}}=\frac{\sigma_{a,\text{int},\text{sl}}}{\sigma_{P_{h}}^{2}}\;,$$ (5)

where $\sigma_{P_{h}}^{2}$ is the phenotypic variance of BW in the selection environment *h*. This value is equal to $\sigma_{P,\text{int}}^{2}$ described above.

Finally, to understand the potential genetic response in ES, the accuracy (*r*_IH_) of predicting breeding value for ES when a selection criterion is BW in one of the environments is equal to:

$$r_{\text{IH}}=\sqrt{\frac{b\text{g}}{\sigma_{a,\text{sl}}^{2}}}=\sqrt{\frac{{\left(\sigma_{\text{a},\text{int},\;\text{sl}}+X\sigma_{a,sl}^{2}\right)}^{2}}{\sigma_{P}^{2}\sigma_{a,\text{sl}}^{2}}}=\frac{\sigma_{a,\text{int},\;\text{sl}}+X\sigma_{a,\text{sl}}^{2}}{\sigma_{P}\sigma_{a,\text{sl}}}\;,$$ (6)

where g is $\sigma_{a,\text{int},\text{sl}}+X\sigma_{a,\text{sl}}^{2}$. The Eq (6) is equivalent to the equation derived by Kolmodin and Bijma [29].

Coheritability of ES changes depending on a degree and forms of GxE. To demonstrate the relationship between coheritability and GxE in both forms, i.e. genotype re-ranking and heterogeneity of variances, Eq (5) is rearranged as (see S1 Appendix):

$$b=\frac{\sigma_{a{,}_{{(\text{E}}_{1},{\text{E}}_{2})}}-\sigma_{a,\text{int}}^{2}}{\sigma_{P,\text{int}}^{2}}\;=\;\frac{r_{g}\sigma_{a,E_{1}}\sigma_{a,E_{2}}-\sigma_{a,\text{int}}^{2}}{\sigma_{P,\text{int}}^{2}}\;,$$ (7)

where *r*_g_ is the genetic correlation between traits measured in two different environments (*E*_1_ and *E*_2_). The *r*_g_ different from unity indicates a present of genotype re-ranking. The $r_{g}=\frac{\sigma_{a{,}_{{(\text{E}}_{1},{\text{E}}_{2})}}}{\sigma_{a,E_{1}}\sigma_{a,E_{2}}}$, where $\sigma_{a{,}_{{(\text{E}}_{1},{\text{E}}_{2})}}$, $\sigma_{a,E_{1}}$ and $\sigma_{a,E_{2}}$ are additive genetic covariance and additive genetic standard deviation in *E*_1_ and *E*_2_, respectively.

Assume that there is no heterogeneity of additive genetic variances ($\sigma^{2}{}_{a,E_{1}}$ = $\sigma^{2}{}_{a,E_{2}}$ = $\sigma_{a,\text{int}}^{2}$) and $\sigma^{2}{}_{P,E_{1}}$ = $\sigma^{2}{}_{P,E_{2}}$ = $\sigma_{P,\text{int}}^{2}$, Eq (7) is equal to:

$$b\;=\;h^{2}r_{g}-h^{2}\;=\;h^{2}\left(r_{g}-1\right)$$ (8)

**Fig 1:**
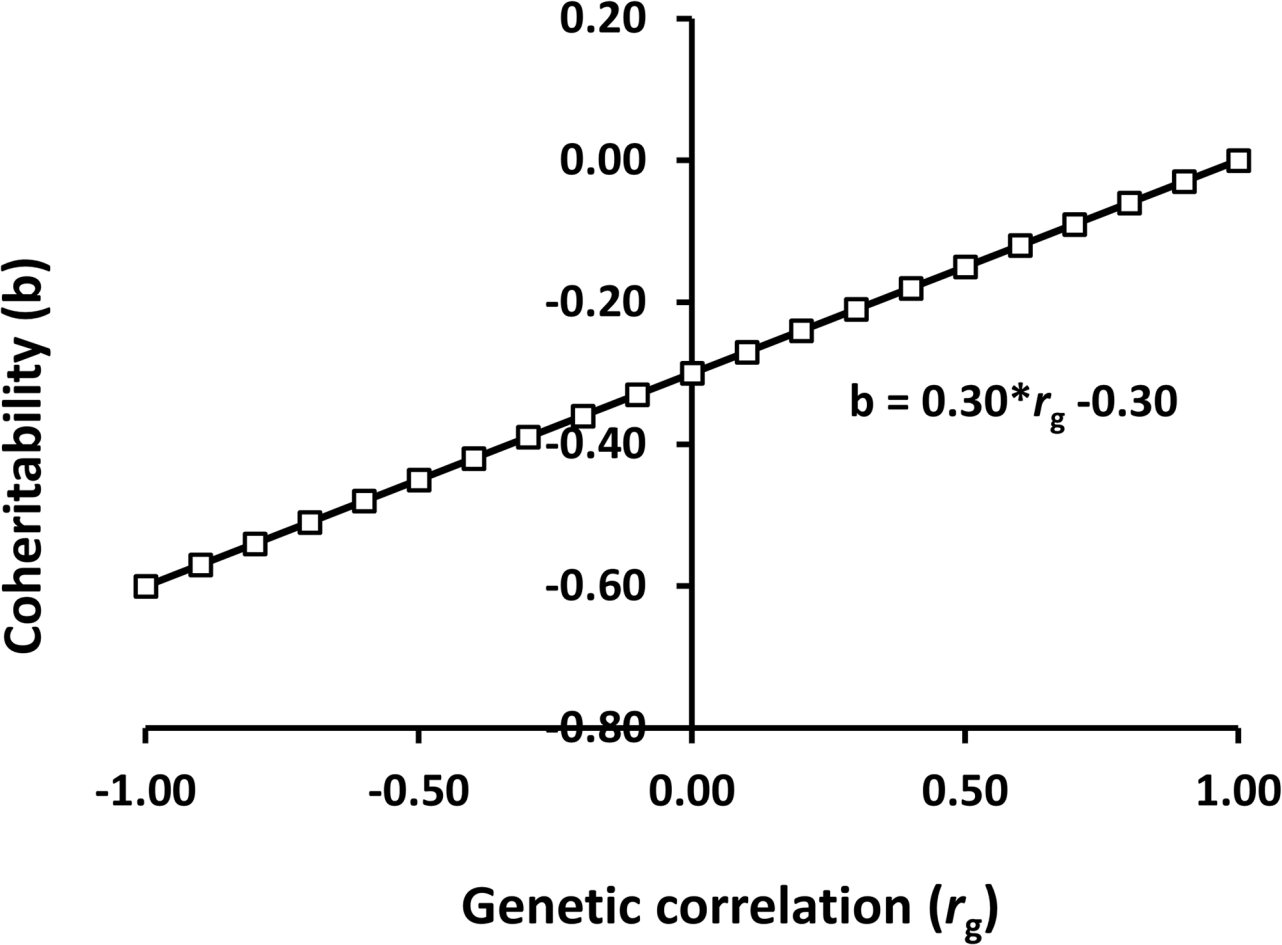
Relationship between coheritability and the genetic correlation between environments. The input parameters are a trait with phenotypic variance of 1 and heritability of 0.3 which are the same across two environments. The genetic correlation ranges from -1 to 1.

Eq (8) shows regression of coheritability on genotype re-ranking, where the slope and intercept is equal to the *h* ^2^ of a trait. If the *h* ^2^ = 0.3 and *r*_g_ varies from -1 to 1, the magnitude of coheritability, regardless of the sign increases when the genetic correlation differs from the unity and the coheritability is at maximum when the genetic correlation equals -1. Placing the intercept (*X* = 0) in either *E*_1_ or *E*_2_ does not influence the magnitude of the coheritability (Fig 1).

Assume there is heterogeneity of additive genetic variances ($\sigma^{2}{}_{a,E_{1}}\neq \sigma^{2}{}_{a,E_{2}}$), Eq (7) is equal to:

$$b\;=\;(h_{E_{1}}h_{E_{2}})r_{g}-h^{2}{}_{\text{int}}$$ (9)

**Fig 2:**
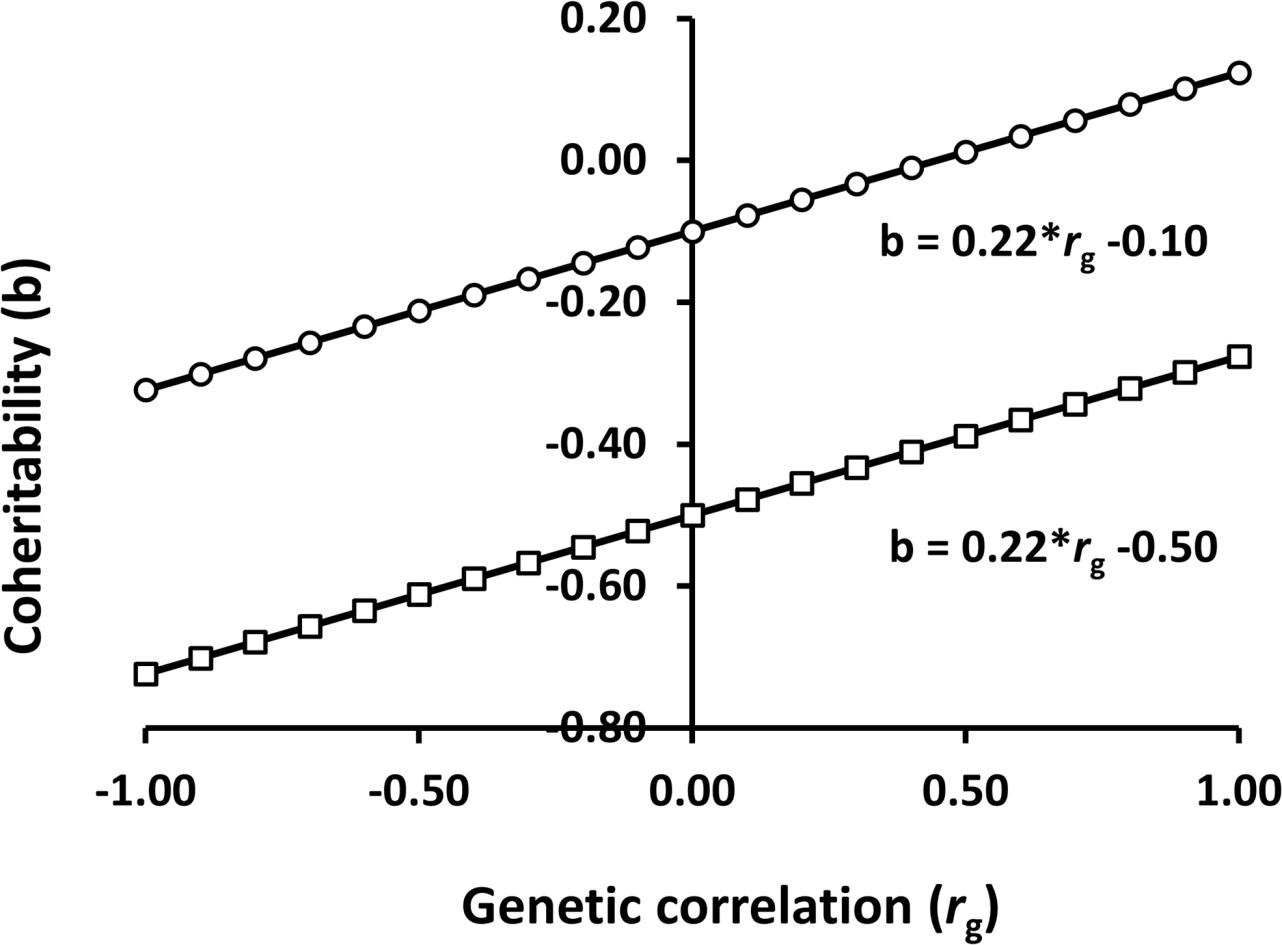
Relationship between coheritability, heterogeneity of additive genetic variances and the genetic correlation between environments. The input parameters are a trait that has different magnitudes of heritability; 0.1 (line with circles) and 0.5 (line with squares) in two different environments (*E*_1_ or *E*_2_) and phenotypic variances are equal to 1. The genetic correlation ranged from -1 to 1. Line graphs show that heterogeneity of additive genetic variances results in different values of coheritability because *h* ^2^_int_ (0.1 or 0.5) changes, depending on the intercept which is placed in either *E*_1_ or *E*_2_.

In contrast to Eq (8), Eq (9) shows that heterogeneity of additive genetic variances results in different values of coheritability because *h* ^2^_int_ changes, depending on the intercept (*X* = 0) which is placed in either *E*_1_ or *E*_2_ as shown in Fig 2.

### Calculation of reaction norm parameters

For the intercept of reaction norms (body weight at the intercept environment), heritability ($h_{\text{int}}^{2}$) and common environmental effect ($c_{\text{int}}^{2}$) were calculated as: $h_{\text{int}}^{2}={\hat{\sigma }}_{a,\text{int}}^{2}/{\hat{\sigma }}_{P,\text{int}}^{2}$, $c_{\text{int}}^{2}={\hat{\sigma }}_{c,\text{int}}^{2}/{\hat{\sigma }}_{P,\text{int}}^{2}$, where ${\hat{\sigma }}_{P,\text{int}}^{2}$ is equal to ${\hat{\sigma }}_{a,\text{int}}^{2}+{\hat{\sigma }}_{c,\text{int}}^{2}+{\hat{\sigma }}_{e,\text{int}}^{2}$, which is the phenotypic variance of BW in the intercept environment when *X* = 0. For the slope of reaction norms, the $h_{ES}^{2}$ was calculated using Eq (1) while the coheritability was calculated using Eq (5), assuming that intercept is placed in the selection environment (*X* = 0) as it reflects actual situation of selective breeding in aquaculture. The coheritability was calculated twice, either having BE or PE as the selection environment. The genetic correlation between intercept and slope (*r*_g(int, sl)_) was calculated as: $r_{\text{g}(\text{int},\;\text{sl})}=\frac{{\hat{\sigma }}_{a_{\text{int},\text{sl}}}}{\sqrt{{\hat{\sigma }}_{a,\;\text{int}}^{2}\times {\hat{\sigma }}_{a,\;\text{sl}}^{2}}}$.

#### Comparison to previous studies

There are no previous studies using reaction norm model to study environmental sensitivity in aquaculture. Hence, to compare the reaction norm parameters of the present study to the previous GxE studies, (co)variance components of the previous studies calculated using multi-trait model were used to calculate the (co)variance components for reaction norm parameters (see S1 and S2 Appendixes). The $h_{ES}^{2}$, coheritability and *r*_g,(int,sl)_ were calculated. The choice of GxE papers in aquaculture species was based on the following: growth traits as the studied trait, at least 30 full-sib families, and providing all the parameters needed for the calculations. In total, 17 studies were found, the species covering Arctic charr (*Salvelinus alpinus*) [30], Atlantic cod (*Gadus morhua*) [31], Common carp (*Cyprinus carpio*) [32], European whitefish (*Coregonus lavaretus*) [33], European sea bass (*Dicentrarchus labrax*) [34], Nile tilapia (*Oreochromis niloticus*) [35,36], Pacific white shrimp (*Litopenaeus vannamei*) [37], Rainbow trout [14,16–21,38], Shiranus tilapia (*Oreochromis shiranus*) [39].

## Results

In the Finnish data, GxE of BW existed in both forms; re-ranking as indicating by *r*_g_ of BW between BE and PE was 0.73, and heterogeneity of genetic variances (Table 2). Both phenomena induce genetic variation for ES.

**Table 2:** Variance components and genetic correlations between intercept and slope from the reaction norm (RN) models.

Parameter	Intercept	
	Production	Breeding
Body weight		
${\hat{\sigma }}_{a,\text{int}}^{2}$	16754.9	18040.0
${\hat{\sigma }}_{a,sl}^{2}$	9584.3	9584.3
${\hat{\sigma }}_{c,\text{int}}^{2}$	3041.1	3227.3
${\hat{\sigma }}_{c,sl}^{2}$	3822.7	3822.7
${\hat{\sigma }}_{e,\text{int}}^{2}$	53197.8	51092.8
${\hat{\sigma }}_{P,Total}^{2}$	73168.5	73168.5
$h_{\text{int}}^{2}$	0.23 (0.03)	0.25 (0.03)
$h_{ES}^{2}$	0.07 (0.03)	0.07 (0.03)
$c_{\text{int}}^{2}$	0.04 (0.01)	0.04 (0.01)
Coheritability	-0.06 (0.02)	-0.08 (0.02)
*r*_*g*(int, *sl*)_	-0.33 (0.10)	-0.41 (0.10)
Log(body weight)		
${\hat{\sigma }}_{a,\text{int}}^{2}$	0.016	0.017
${\hat{\sigma }}_{a,sl}^{2}$	0.011	0.011
${\hat{\sigma }}_{c,\text{int}}^{2}$	0.003	0.003
${\hat{\sigma }}_{c,sl}^{2}$	0.004	0.004
${\hat{\sigma }}_{e,\text{int}}^{2}$	0.092	0.071
${\hat{\sigma }}_{P,Total}^{2}$	0.102	0.102
$h_{\text{int}}^{2}$	0.15 (0.02)	0.18 (0.03)
$h_{ES}^{2}$	0.06 (0.02)	0.06 (0.02)
$c_{\text{int}}^{2}$	0.03 (0.01)	0.04 (0.01)
*r*_*g*(int, *sl*)_	-0.40 (0.10)	-0.42 (0.10)

### Genetic variance for macroenvironmental sensitivity

The additive genetic variance of slope of BW (9584) was considerable and the $h_{\text{int}}^{2}$ was moderate in both environments (0.23 for PE and 0.25 for BE), the $h_{ES}^{2}$ was low (0.07) implying the additive genetic variance of ES explains only a small proportion relative to total phenotypic variance of BW across environments. Similarly, the coheritability for ES was low and negative, i.e., -0.06 for PE and -0.08 for BE. Thus the accuracy of selection for ES of BW is very low when applying individual selection for BW in one of the environments.

The magnitude of heritability for ES of log-transformed BW was similar to the heritability for ES of observed BW. The additive genetic variance of slope of log-transformed BW was 69% in PE and 65% in BE of the additive genetic variance of intercept, relatively slightly higher than on the observed scale (57% in PE and 53% in BE). This indicates that simple scale effects did not generate genetic variation for macroenvironmental sensitivity.

**Fig 3:**
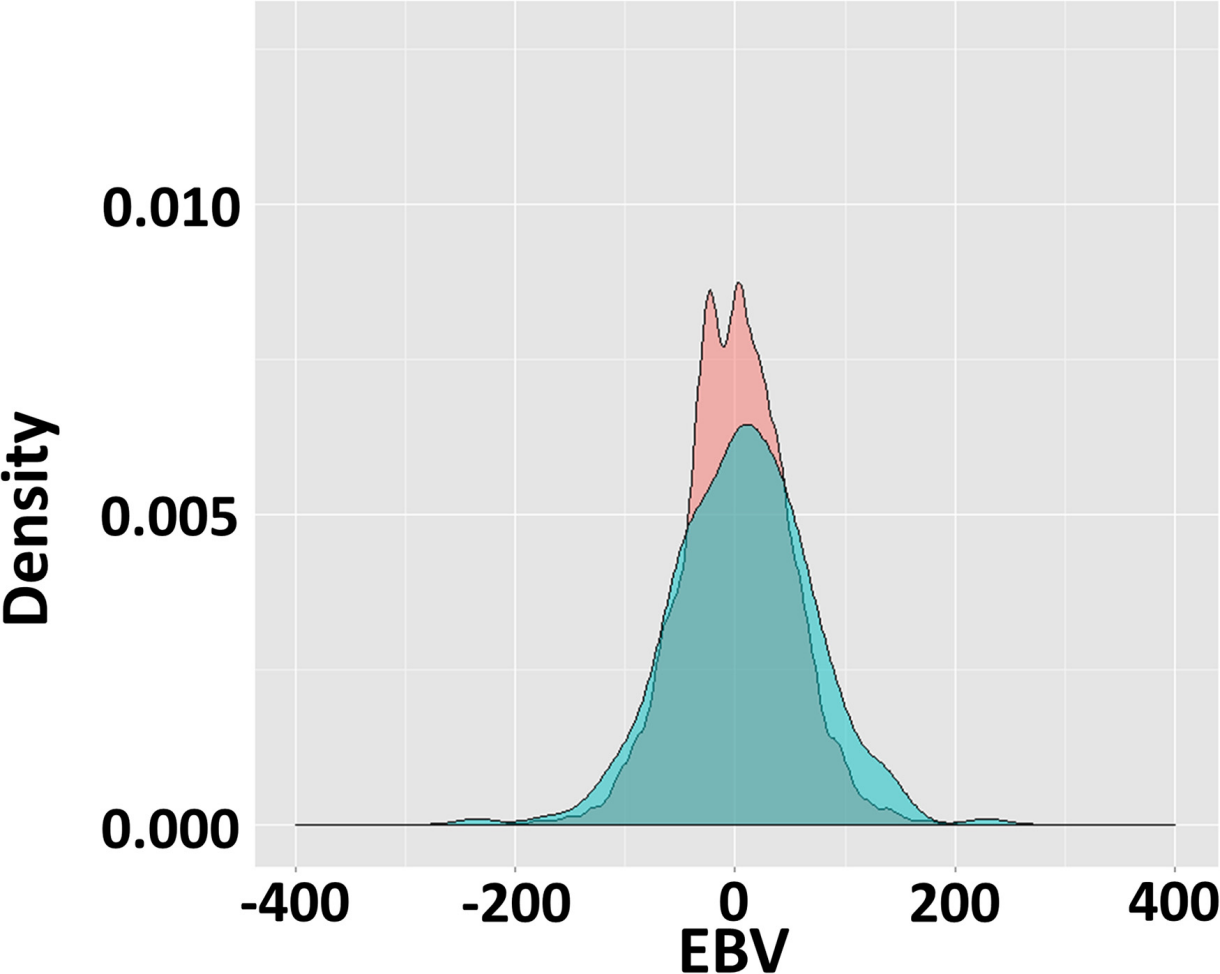
Probability density distribution of estimated breeding value (EBV) of macroenvironmental sensitivity. The EBV estimated using reaction norm model where the intercept was production environment. Light green colour is the EBV distribution of sire whereas light red colour is the EBV distribution of sire’s offspring.

When PE was the intercept, the slope EBVs for sires (-234.5 to 228.8) and animals (-210.6 to 199.8) ranged from strongly negative to positive (Fig 3). A positive slope implies that EBVs for BW are elevated in BE as compared to EBVs for BW in the intercept environment PE. If BE was the intercept environment, the EBVs of slope would change sign.

### Genetic correlation between intercept and slope

**Fig 4:**
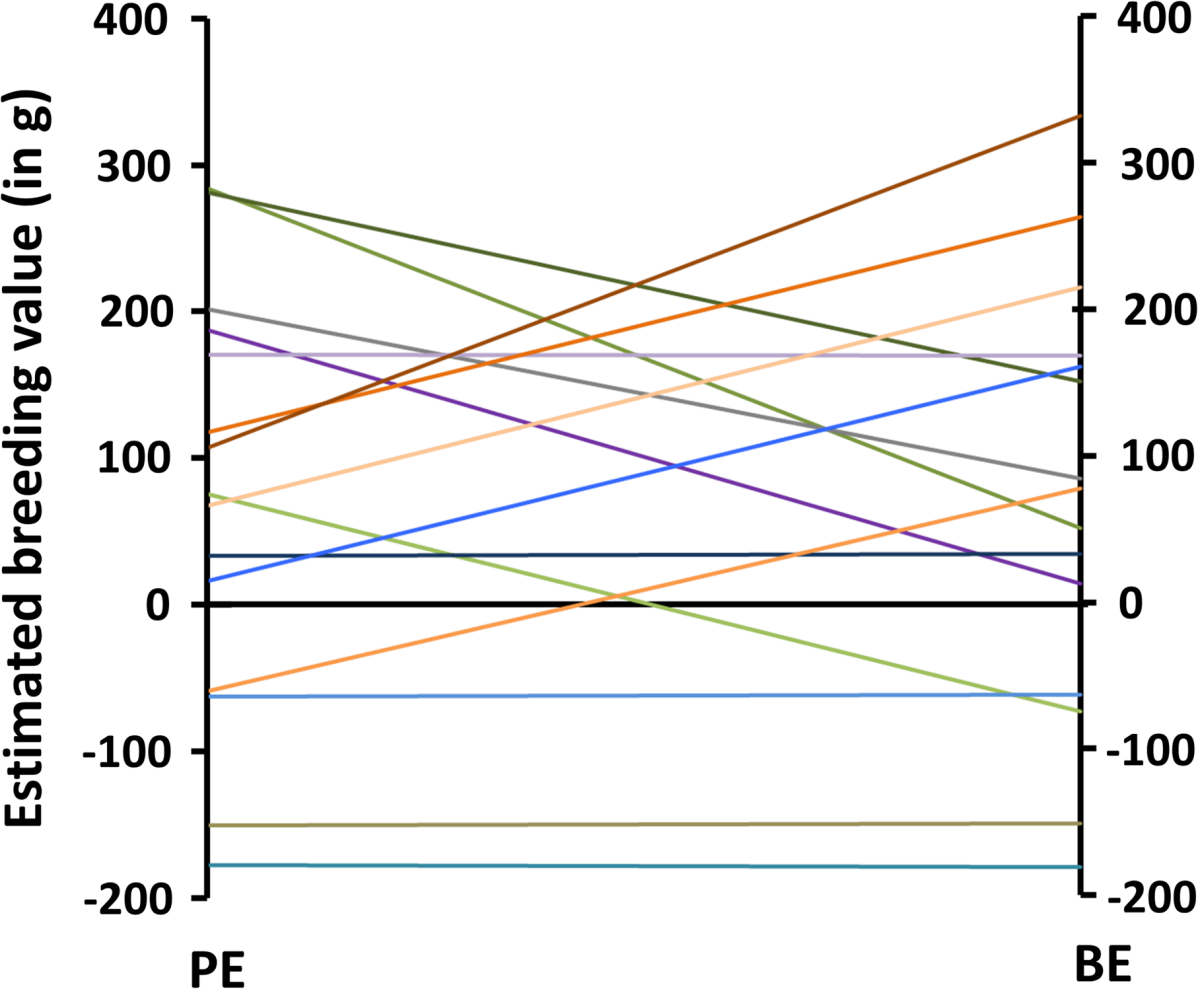
Reaction norm of sires across production (PE) and breeding (BE) environments. A total number of fifteen sires were chosen from highest, close to zero and lowest EBVs for the slope. The intercept is placed at PE (*X* = 0).

The significant *r*_*g*(int, sl)_ (SE) between BW in a given environment and ES ranged from -0.33 (0.10) to -0.41 (0.10), depending on the environment used as the intercept environment (Table 2). The sires with steep slope EBVs had high intercept (at PE or BE) (Fig 4). The sires with flat slope EBV had low intercept EBV. The negative correlations from log-transformed data (-0.40 to -0.42) remained similar to untransformed data. This shows that rapid growth in one environment is genetically related to elevated sensitivity across environments.

### Genetic parameters calculated from the previous GxE studies

**Fig 5:**
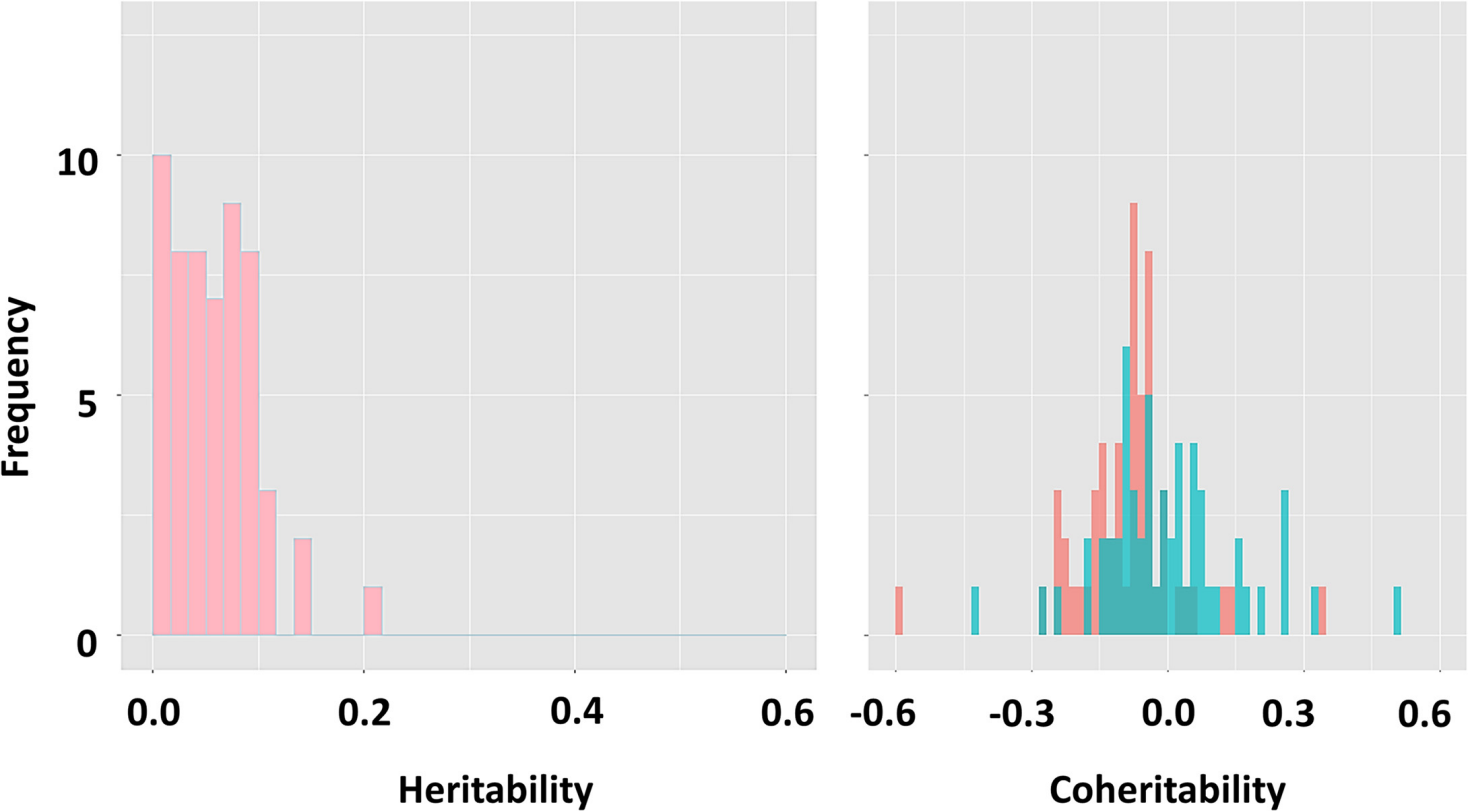
Histogram of heritability and coheritability for macroenvironmental sensitivity of growth traits obtained from previous GxE studies in aquaculture. The coheritability differs in different environments. The light red and light green bars show two distributions of coheritability in two different environments. The dark green bar indicates the position that two histograms overlap.

**Fig 6:**
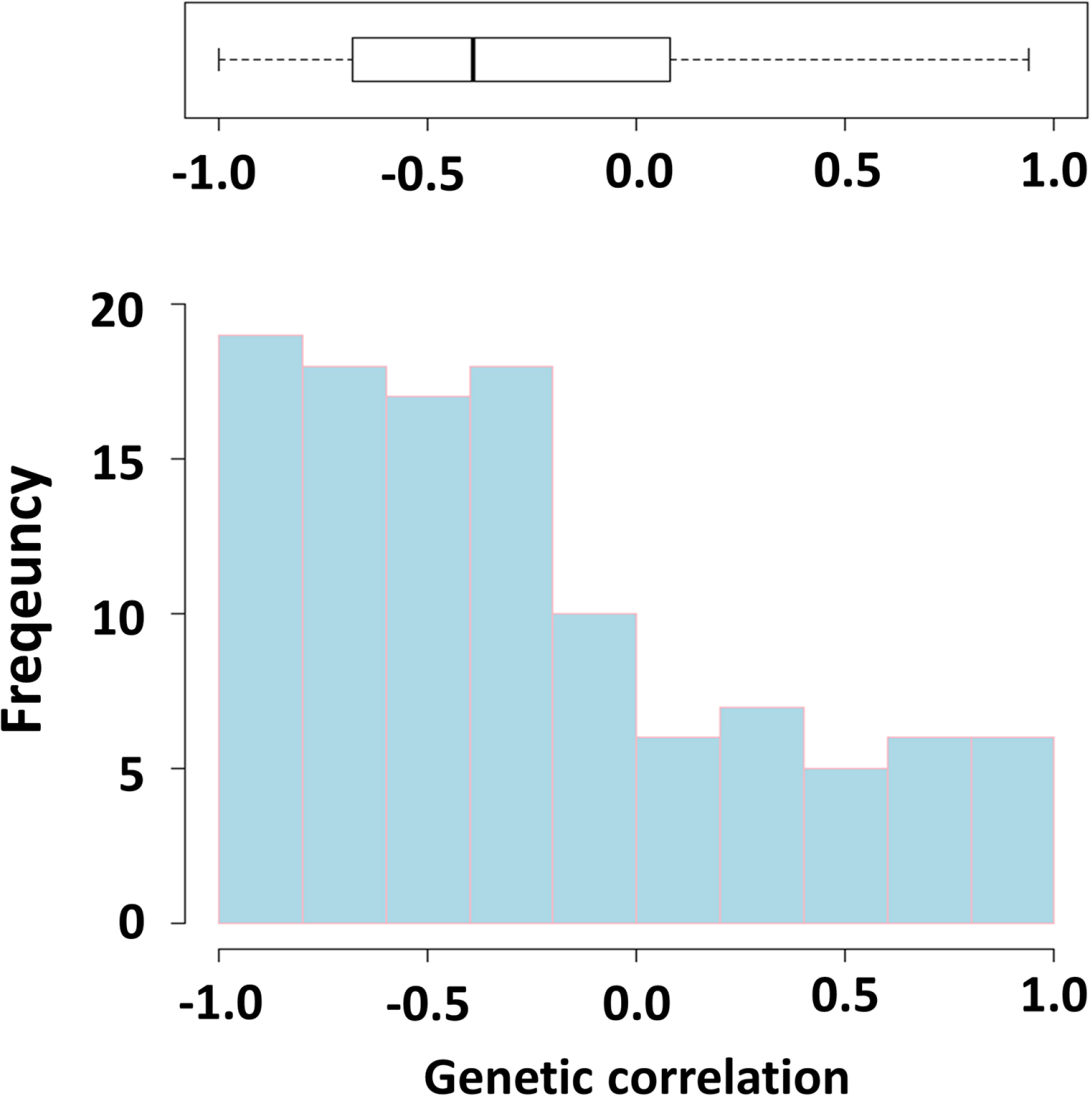
Histogram and box plot of genetic correlation between intercept and reaction norm slope of growth traits (*r*_g(int,sl)_). The parameters estimated were obtained from previous GxE studies in aquaculture.

In the previous aquaculture studies on GxE in growth, the $h_{ES}^{2}$ ranged from 0.010 to 0.207 (median = 0.110) while the coheritability ranged from -0.600 to 0.500 (median = -0.011; intercept at *E*_1_ and = -0.078; intercept at *E*_2_) (Fig 5). The *r*_*g*(int, sl)_ between growth traits and ES varied from -1.00 to 0.94 (median = -0.386) as shown in Fig 6.

## Discussion

### Genetic variation for macroenvironmental sensitivity

Substantial additive genetic variance of macroenvironmental sensitivity (ES) for both observed and log-transformed body weight (BW) indicates potential for genetic response to selection on ES. After the log-transformation of BW, the variance components of ES were reduced but $h_{ES}^{2}$ remained similar to the one estimated from the untransformed data. This indicates that scale effects (high variance depending on high mean) do not explain the genetic effects for ES.

The $h_{ES}^{2}$ introduced by Scheiner [27] is a descriptive parameter indicating that ES has a heritable component but it is not a parameter to infer the accuracy of selection for ES. In most cases, in aquaculture species, individuals do not have a phenotype for ES. The coheritability explains heritable genetic variance of ES of BW when the selection criterion is BW in one environment. In contrast to $h_{ES}^{2}$, coheritability changes when the selection environment changes because BW in the BE and PE are genetically different traits. Although the sign of coheritability is the sign of the correlated response [13], the coheritability differs from the genetic correlation because the genetic correlation does not reflect the accuracy of selection [40].

Despite the presence of additive genetic variance for ES, low $h_{ES}^{2}$ (0.07) infers that the additive genetic variance of ES explains only a small proportion of the total phenotypic variance of BW across the environments. Our finding is consistent with the previous studies, showing that $h_{ES}^{2}$ is generally low. In our study, the amount of genetic variance for the slope varies from 53 to 57% of the genetic variance of BW, which is among the highest (5 to 60%) in a review by Scheiner [24]. The coheritability of -0.08 for BE and of -0.06 for PE, suggested that a single BW observation results in low accuracy of selection for ES. Because multi-trait and reaction norm models are interchangeable (see S1 and S2 Appendixes), it is possible to obtain genetic parameters of ES from the previous GxE studies of growth traits in aquaculture [14,16–21,30–39]. Our estimates of $h_{ES}^{2}$ are within the range of previous studies (${\bar{h}}_{ES}^{2}$ = 0.058: min = 0.010 and max = 0.207). Similarly, the coheritability estimated in the present study is in line with the previous studies (mean = -0.047: min = -0.600 and max = 0.500) [14,16–21,30–39]. In the literature, the most extreme values of the (co)heritability (0.415 for $h_{ES}^{2}$; -0.600 for coheritability) were found for Nile tilapia with strong re-ranking of growth (*r*_g_ = -0.27) and the presence of heterogeneity of genetic variance (${\hat{\sigma }}_{a,E_{1}}^{2}$ = 0.265 and ${\hat{\sigma }}_{a,E_{2}}^{2}$ = 0.001) [35]. This clearly shows that the magnitude of (co)heritability increases, regardless the sign, when GxE is larger.

To gain more understanding of potential for a change in sensitivity, we calculate accuracy of selection by using Komoldin and Bijma’s equation ($\frac{{\text{r}}_{\text{g}(\text{int},\text{sl})}{\hat{\sigma }}_{a,\;\text{int}}+X{\hat{\sigma }}_{a,\;\text{sl}}}{{\hat{\sigma }}_{P_{\text{h}}}}$) [29]. For instance, mass selection for BW is practiced within many broodstocks of rainbow trout. When BW in PE or BE is the selection criterion, the accuracy of selection for sensitivity is either -0.158 or -0.205, respectively. The low accuracies are logical because in our data, the reaction norm slope was not measured from an individual itself. In our data, the breeding value for the slope can only be estimated by using BW records from relatives. For sires and dams with offspring in both environments, the slope EBVs are likely to be recorded with moderate-to-high accuracy. This conclusion is similar to the microenvironmental sensitivity of BW in Atlantic salmon and rainbow trout for which the phenotype of an individual (squared residual) poorly predicts the breeding value of uniformity of BW [41–44]. When only a single BW record exists for an individual, macroenvironmental and microevironmental sensitivities can only be recorded from groups of individuals. Naturally, for a breeding programme with pedigreed population and performance testing in several environments, selection can be directly practiced on slope EBVs, or ES can be controlled by using appropriate index weights for environment-specific EBVs of BW.

### Body weight and environmental sensitivity

The genetic correlation between slope and intercept is negative when the intercept is placed either in BE (-0.41) or PE (-0.33). In other words, in our data, placing the intercept in one or the other environments has no large effect on the magnitude of genetic correlation between intercept and slope. Hence, selection for fast growth in one environment will lead to increased sensitivity, i.e., to steeper negative slopes. The genetic correlation is low enough that both traits can be improved simultaneously. However, this would compromise the genetic gain in the selected environment. When looking at the sires with the most extreme EBVs for slope, the sires with flat slope EBVs have low overall growth in both environments (Fig 4). It is unknown why the fastest growing genotypes are more sensitive, but it may be related to different sets of genes controlling growth in different environments.

Although, the genetic correlations in this study are negative, the sign of genetic correlation will be the opposite (from negative to positive) when setting the environmental variable for example to -1 and 0. The pros and cons of the reaction norm model are detailed by Sae-Lim et al. [45]. Considering the absolute genetic correlations, the conclusion that can be drawn is that BW in one environment is genetically related to ES of rainbow trout population, in line with the estimates from previous aquaculture studies (median = 0.573) [14,16–21,30–39].

It is possible to gain additional understanding of ES in rainbow trout by considering Jinks-Connolly’s rule [8,46] when there are two environments. Jinks and Connolly [46] suggested that *antagonistic selection*, i.e., selection for high value of phenotype in an environment that has a lower phenotype comparing to another environment, reduces ES, while *synergistic selection*, i.e., selection for high value of phenotype in an environment that has a higher phenotype comparing to another environment, increases ES. Falconer's review [8,47] proved this to be generally true in 14 to 16 out of 21 cases. In the Finnish breeding programme for rainbow trout, it is expected that ES is increased both when selecting on PE or BE because the mean BWs are in fact very similar in both environments. Hence, in both environments, selection in either environment will lead to higher direct genetic response compared to the correlated genetic response in the other environment. This is not in contrast to Jinks-Connolly rule but it is a consequence that not one of the environments support superior mean BW. In the practical breeding programme for Finnish rainbow trout, the selection index puts more weight at BW in PE (85%) than BW in BE (15%), implying that the expected change in ES (if selecting only in PE) will be reduced by giving index weight to both BW in BE and PE.

In some breeding programmes, BW is selected in two environments to improve mean performance of BW over the two environments. However, there may be a trade-off between selection for increased mean BW and decreased ES [47]. To understand the trade-off between selection for increased mean performance and reduced environmental sensitivity (increased stability), we calculate the genetic correlation between mean performance and sensitivity using the equation derived by Rosielle and Hamblin [47,48]: $\frac{K_{G}-1}{\sqrt{1+2K_{G}^{2}+K_{G}^{4}-4r_{g}^{2}K_{G}^{2}}}$. The *K*_G_ is the ratio of the additive genetic variances of a trait in high-performing environment to low-performing environment. In the presence data, BE is the high-performing environment. Hence, the *K*_G_ can be calculated as 16754.9/18040 = 0.93. Substituting *K*_G_ (0.93) and *r*_g_ (0.73) into the equation results in the genetic correlation between mean performance and sensitivity of -0.11. Hence, selection for increased mean performance of BW or the current selection index may increase sensitivity in Finnish rainbow trout population across the two environments, a conclusion similar to if selection is practiced only in one of the environments.

It may be interesting to limit such trade-off by implementing restricted selection on environmental sensitivity, i.e., zero change in environmental sensitivity while selecting for high phenotype. In case of 2 environments, it is possible to maintain genetic gains of BW in two environments and thus ES is maintained stable. Alternatively, if the selection index includes mean BW across environments and sensitivity [47], selection index weights producing desired genetic gains can be obtained using the formula developed by Brascamp [49]. In case of a continuous environmental variable, Komoldin and Bijma [29] have derived the equation: $-\;{\text{r}}_{\text{g}(\text{int},\;\text{sl})}\frac{{\hat{\sigma }}_{a,\;\text{int}}}{{\hat{\sigma }}_{a,sl}}$ to determine the level of environmental factor in which selection on a phenotype will result in no change in environmental sensitivity. For instance, the environmental level where there is no change in sensitivity,-(-0.967*0.302/0.019) = 15°C using genetic covariance matrix from a study on age at maturity of *Daphnia galeata* at three temperature levels, 10, 15 and 20°C [23]. This temperature level of 15°C corresponds to the zero genetic covariance between intercept and slope when the intercept is set to 15°C [23]. Interestingly, this information could be utilized to determine environmental level for simultaneous selection for high overall phenotypic value and for no change in environmental sensitivity.

The future research may continue to identify responsible environmental variables explaining GxE between environments [50]. Subsequently, environmental level, corresponding to no change in sensitivity of rainbow trout body weight can be determined using Kolmoldin and Bijma’s equation. Selection for improved stability of performance may also be interesting for shrimp breeding. For example, stability of growth in low level of salinity is important for inland marine shrimp farming, because soil salinization is a potential environmental impact for agriculture [51].

### Increased macroenvironmental sensitivity: good or bad?

In animal breeding, reduced environmental sensitivity is considered to be beneficial in many aspects. First, it improves stability of animal performance across environments. Farmed fish being well adapted to multiple environments and variable environmental conditions may increase overall survival and animal welfare. Secondly, this simultaneously increases aquaculture industry profit because stability in fish performance may lead to on average higher production. However, the opposite is arguable. First, farmed animal performances are expected to respond positively when an environmental variable is improved. For example, fish are expected to grow faster when quality of feed is improved. Second, if a mean performance of a fish stock or a genotype is low and the genotype does not respond to any change across environments, it may not be desirable for a fish breeder because fish will perform poorly across environments [46]. Positive response as the environmental changes may be useful for developing a locally-adapted population for that environment. Hence, macroenvironmental sensitivity may be viewed as both an opportunity and a challenge for selective breeding [46].

## Conclusions

Genetic changes in macroenvironmental sensitivity of body weight in rainbow trout can be expected due to high additive genetic variance. Macroenvironmental sensitivity is increased when selecting for high body weight in either environment. To avoid an increase of macroenvironmental sensitivity while selecting for body weight, it is possible to maintain fixed genetic gains of body weight in two environments and thus macroenvironmental sensitivity is maintained stable. Alternatively, if the selection index includes mean body weight across environment and macroenvironmental sensitivity, selection index weights producing desired genetic gains should be implemented.

## Supporting Information

S1 Appendix Interchangeable genetic covariance matrices. (DOCX)

S2 Appendix Calculation of reaction norm genetic covariance matrix (G_RN_). (DOCX)
