# Supplementary material for: Genetics of Growth Reaction Norms in Farmed Rainbow Trout
Source: PLoS One. 2015 Aug 12;10(8):e0135133. doi: 10.1371/journal.pone.0135133 (PMC4534094; doi:10.1371/journal.pone.0135133)
Supplement: S1 Appendix — (DOCX) [file pone.0135133.s001.docx]

# Supporting Information

# S1 Appendix. Interchangeable genetic covariance matrices. In this appendix, we describe the equations used to obtain reaction norm parameters from multi-trait model. The multi-trait model was:

$y_{hijklmn}=\mu+YC\times SITE\times SEX\times MAT_{ijkl}+a_{hm}+t_{in}+e_{hijklmn}$,

where *y* is body weight of the *m*^th^ individual in a given environment (*h* =1: PE, 2: BE), *µ* is the overall mean, the $a_{hm}$ is the random additive genetic effect, *a ~* MVN[**0**, **A****G**_MUV_ ], **G**_MUV_ is the additive genetic (co)variances matrix. The residual covariance of the BW measured in different environments was set to zero because each animal lived in only one environment.

When the dimension of the genetic covariance matrix and the fixed effects included in a multi-trait and a reaction norm models are the same, genetic covariance matrices between the multi-trait and reaction norm models are interchangeable [7,22]. Hence, the breeding value (*a*) for the slope (sl) can be derived as a change of the two breeding values estimated from the multi-trait model when the differences between *X* is equal to 1:

$a_{sl}=\left(a_{\text{BW}_{\text{BE}}}-a_{\text{BW}_{\text{PE}}}\right)$ , (1)

where$a_{\text{BW}_{\text{BE}}}$,$a_{\text{BW}_{\text{PE}}}$are additive genetic for body weight (BW) in breeding (BE) and production (PE) environments, respectively. The additive genetic variance of slope ($\hat{\sigma}_{a,\text{sl}}^{2}$) is equal to:

$$\hat{\sigma}_{a,\text{sl}}^{2}=\mathrm{var}\left(a_{\text{BW}_{\text{BE}}}-a_{\text{BW}_{\text{PE}}}\right)$$

(2)

To obtain the genetic covariance between intercept and slope, we first use the covariance function in a reaction norm model to calculate the additive genetic covariance of BW between BE and PE:

This will result in the linear combination of

,

which can be rearranged to

**(**3**)**

When the$X_{h}$ is equal to a theoretical intercept, i.e., $X_{h}$= 0, the$\hat{\sigma}_{a,\text{ int}}^{2}$is equal to additive genetic variance of a trait in the environment assigned as the intercept ($\hat{\sigma}_{a,\text{ }h=\text{int}}^{2}$). Therefore, it is possible to substitute $\hat{\sigma}_{a,\text{ }h=\text{int}}^{2}$to the equation.

(4)

In case of $X_{h_{1}}$and $X_{h_{2}}$are 0 and 1, the equation can be simplified to:

(5)

If the intercept is moved, the $\hat{\sigma}_{a,\text{ int}}^{2}$will deviate from$\hat{\sigma}_{a,h=\text{int}}^{2}$(see Tienderen and Koelewijn [23]). Consequently, substituting $\hat{\sigma}_{a,\text{ }h=\text{int}}^{2}$obtained from the multi-trait model into the equation will not result in the$\hat{\sigma}_{a _{\mathrm{int},\text{sl}}}$from the reaction norm model.
