# Supplementary material for: Genetics of Growth Reaction Norms in Farmed Rainbow Trout
Source: PLoS One. 2015 Aug 12;10(8):e0135133. doi: 10.1371/journal.pone.0135133 (PMC4534094; doi:10.1371/journal.pone.0135133)
Supplement: S2 Appendix — (DOCX) [file pone.0135133.s002.docx]

# Supporting Information

# S2 Appendix. **Calculation of reaction norm genetic covariance matrix (G_RN_).**

|  |  |  |
| --- | --- | --- |
| (a) Genetic covariance matrix from the multi-trait model (**G**_MUV_) | | |
| Environment | Breeding | Production |
| Breeding | 18040.0 |  |
| Production | 12605.3 | 16754.9 |
|  |  |  |
| (b) Intercept at breeding environment, interchangeable between  **G**_MUV_ and **G**_RN_, | | |
| $\hat{\sigma}_{a,\text{sl}}^{2}$ = 18040.0 + 16754.9 - 2*12605.3 | |  |
| $\hat{\sigma}_{a _{\mathrm{int},\text{sl}}}$= 12605.3 - 18040.0 | |  |
|  |  |  |
|  | Intercept | Slope |
| Intercept | 18040.0 |  |
| Slope | -5434.7 | 9584.3 |
|  |  |  |
| (c) Intercept at production environment, interchangeable between  **G**_MUV_ and **G**_RN_, | | |
| $\hat{\sigma}_{a,\text{sl}}^{2}$ = 18040.0 + 16754.9 - 2*12605.3 | |  |
| $\hat{\sigma}_{a _{\mathrm{int},\text{sl}}}$= 12605.3 - 16754.9 | |  |
|  |  |  |
|  | Intercept | Slope |
| Intercept | 16754.9 |  |
| Slope | -4149.6 | 9584.3 |
